# Association of *RAC1* Gene Polymorphisms with Primary End-Stage Renal Disease in Chinese Renal Recipients

**DOI:** 10.1371/journal.pone.0148270

**Published:** 2016-02-03

**Authors:** Yani Liu, Jiali Zhou, Xiaomei Luo, Chunxiao Yang, Yu Zhang, Shaojun Shi

**Affiliations:** Department of Pharmacy, Union Hospital, Tongji Medical College, Huazhong University of Science and Technology, Wuhan, China; University of Birmingham, UNITED KINGDOM

## Abstract

**Background/Objective:**

*RAC1* gene could influence susceptibility to renal failure by altering the activity and expression of Rac1, which is a member of the Rho family of small GTP-binding proteins. In clinical practice, renal transplantation provides the optimal treatment for people with end-stage renal disease (ESRD). The objective of this present study was to determine whether the *RAC1* gene polymorphisms were associated with primary ESRD susceptibility in Chinese renal recipients.

**Methods:**

Six single nucleotide polymorphisms (SNPs) of *RAC1* gene, including rs836488 T>C, rs702482 A>T, rs10951982 G>A, rs702483 A>G, rs6954996 G>A, and rs9374 G>A, were genotyped in 300 renal transplant recipients (cases) and 998 healthy Chinese subjects (controls) by using TaqMan SNP genotyping assay. Allele, genotype, and haplotype frequencies of the six SNPs were compared between cases and controls. Odds ratios (OR) and 95% confidence intervals (CI) were calculated in logistic regression models to evaluate the associations of the six SNPs with ESRD risk.

**Results:**

The genotype distributions for the six SNPs in controls were consistent with Hardy-Weinberg equilibrium (*P* > 0.05). Association analysis revealed that three SNPs were significantly associated with ESRD risk. Positive associations with ESRD risk were found for the rs836488, rs702482, and rs702483 in the co-dominant model (minor allele homozygotes versus major allele homozygotes); specifically, the frequencies of the minor allele homozygotes and the minor allele for the three SNPs were higher in the cases than in the controls. In addition, these three SNPs also had associations with increased ESRD risk under the additive model (*P* < 0.05), and positive associations were also found for the rs836488 in the dominant model (*P* < 0.05) and for the rs702483 in the recessive model (*P* < 0.05). All these associations were independent of confounding factors. The other three SNPs (rs10951982, rs6954996, and rs9374), in all comparison models, were not associated with ESRD risk (*P* > 0.05). In haplotype analysis, carriers with "C-T-G-G-G-G" haplotype had a significantly higher risk of ESRD compared with the most common haplotype "T-A-G-A-G-G" (*P* = 0.011, OR = 1.46, 95% CI = 1.09–1.94).

**Conclusion:**

This study suggested that polymorphisms of *RAC1* gene might influence the susceptibility to ESRD in Chinese Han population. Further studies are necessary to confirm our findings.

## Introduction

End-stage renal disease (ESRD), the complete or almost complete failure of the kidneys to function, has become a worldwide public health problem, with increased risks of mortality and morbidity [1, 2]. In China, the annual incidence of ESRD was estimated to be 36.1 per million population [3], and the number of ESRD patients has been increasing approximately three times over the past decade [4]. Lots of risk factors have been reported to be susceptible to develop rapid progressive ESRD, including immunological and environmental factors [5]. Recently, a growing body of studies have focused on associations of genetic polymorphisms in candidate genes with the risk of ESRD [6–10]. The latest developments in genomewide association studies (GWAS) have identified a number of genetic-variants associated with impairment of renal function [1], including *MECOM*, *UNCX*, *WDR72*, *UMOD*, and *GNAS* [1, 11–13]. Nevertheless, other genes could also be candidates and further assessment is desirable.

The Ras-related C3 botulinumtoxin substrate 1 (Rac1), encoded by the *RAC1* gene, is a member of the Rho family of small guanosine triphosphatases (GTPases) and it cycles between an inactive GDP-bound state and an active GTP-bound state [14]. The structure of human *RAC1* gene, which is located on chromosome 7p22, had been completely described by Matos *et al*. [15]. It has been well-known that Rac1 plays significant roles in regulating various cellular processes, including cell division, proliferation, transformation, differentiation [16, 17], vesicle transport, nuclear assembly, cytoskeletal reorganization, gene transcription [18], survival and motility [17, 19]. Rac1 is also known as a protein controlling reactive oxygen species (ROS) production [20, 21], receptor-associated intracellular signaling [22], and nuclear factor B activation [17].

Nowadays, *RAC1* gene polymorphisms have been proved to be associated with the development of various diseases, such as ovarian cancer [23], breast cancer [24], testicular cancer [24], ulcerative colitis [25], and Crohn’s disease [25, 26]. It was revealed that the polymorphism rs10951982 (G/A) in the intron 1 of *RAC1* gene could increase *RAC1* gene expression, which contributed to increased neutrophil recruitment to the colon and increased proinflammatory cytokine expression in the colonic tissue [25].

Furthermore, several studies have revealed that Rac1 could be associated with the development of cardiovascular damage, which might be due to a crosstalk effect between Rac1 and mineralocorticoid receptor activation independent of aldosterone [27–29]. And according to the previous observations, it has been proposed that alterations of Rac1 could influence susceptibility to diseases such as hypertension or renal failure by impairing the correlated proteins or altering the activity or expression of Rac1 [27, 30]. It is reasonable to speculate that *RAC1* gene might be implicated in hypertension or ERSD. Moreover, it has been demonstrated that SNPs rs836478 (C/T) and rs10951982 (G/A) in *RAC1* gene were associated with higher level of biomarkers (interleukin 6, metalloproteinase-9, and plasminogen activator inhibitor-1) related to the development and progression of hypertension [27]. While to date no study has assessed the associations of *RAC1* genetic variants with susceptibility to ERSD.

Clinically, renal transplantation could evidently improve the survival and quality of life for patients with ESRD as the optimal treatment. To date, however, no study concerning the genetic variation of the *RAC1* gene in renal transplant recipients has been carried out. In order to find out the potential correlation of *RAC1* gene polymorphisms with primary ERSD in renal transplant recipients, the allelic and genotypic frequencies of the six *RAC1* SNPs (rs836488, rs702482, rs10951982, rs702483, rs6954996, and rs9374) in 300 renal transplant recipients and 998 healthy subjects from China were investigated.

## Materials and Methods

### Study population

From November 2012 to March 2013, renal transplant recipients were consecutively recruited from the Wuhan Union Hospital and Tongji Hospital (Wuhan, China), who came to the outpatient departments of the two hospitals for the routine scheduled check-up. Patients with a functioning renal graft for at least six months were eligible, irrespective of the underlying primary renal disease and gender. Patients with multiple organ transplantations, malignancy, human immunodeficiency virus infection, serve cardiac dysfunction, serve pulmonary dysfunction, server hepatic dysfunction, or active infection were excluded. A total of 367 renal transplant recipients were eligible, of whom 304 patients were successfully consented and enrolled. Of those enrolled patients, 4 patients with poor quality DNA samples were excluded. Healthy controls, with no history of renal disease, hypertension, or diabetes, were randomly selected from a pool of healthy volunteers who visited the same hospitals for routine physical examinations at the same time. To control for the effects of potential confounders, controls were frequency matched to cases by age (within 5 years) and gender. A total of 998 healthy controls were included in this study. Demographic characteristics (such as age and gender), lifestyle factors (such as tobacco smoking and alcohol consumption), and medical history (such as hypertension and diabetes) were collected from the interviewer-administered questionnaires. Individuals who smoked more than 100 cigarettes in their lifetime were defined as smokers. Individuals who consumed alcoholic beverages at least three times per week for more than six months in their lifetime were defined as drinkers. All study subjects were self-reported ethnically Han Chinese and genetically unrelated.

The study was approved by the independent ethics committee of Tongji Medical College of Huazhong University of Science and Technology, and all study-related procedures were conducted in accordance with the principles of the Declaration of Helsinki. All participants were informed of the investigational nature of this study and provided written informed consent prior to initiation of any study related procedures.

### SNPs selection

**Fig 1:**
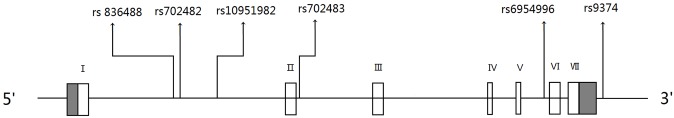
Schematic presentation of the structure of RAC1 gene indicating locations of the analyzed variants (rs836488, rs702482, rs10951982, rs702483, rs6954996, and rs9374). The RAC1 gene consists of 7 exons (I-VII).

First, we screened the dbSNP database of NCBI to select common SNPs that localized within the gene region of *RAC1* (including 2kb upstream and downstream of *RAC1*), with a minor allele frequency (MAF) greater than 0.05 in the CHB population. Then, the SNPinfo Web Server (http://snpinfo.niehs.nih.gov/snpinfo/snpfunc.htm) was applied to filter these SNPs for functional SNPs. Additionally, SNPs that had been previously reported to be associated with some diseases [25, 31, 32] were also selected. Finally, six SNPs were selected for genotyping, which were rs836488, rs702482, rs10951982, rs702483, rs6954996, and rs9374. The locations of the six SNPs in *RAC1* gene are shown in Fig 1.

### DNA extraction

Venous blood samples from participants were collected into EDTA-treated vacutainer tubes. Genomic DNA was extracted from the whole blood and isolated using QIAamp^®^ DNA Blood Mini Kit (Qiagen, Germany) according to the manufacturer’s instructions, and then quantified using a Nanodrop 1000 Spectrophotometer (Thermo Scientific, Wilmington, DE, USA). Extracted DNA samples were stored at −80°C until processing for genotyping analysis.

### Genotyping analysis

The genotypes of the six SNPs (rs836488, rs702482, rs10951982, rs702483, rs6954996, and rs9374) in *RAC1* gene were determined using TaqMan SNP genotyping assay (Applied Biosystems, Foster City, CA, USA). The sequences of the primers for the six SNPs are presented in Table 1. PCR amplification was carried out in a total volume of 10 μL, containing 25 ng DNA, 5 μL TaqMan genotyping master mix, 1.0 μL TaqMan SNP genotyping assay mix, and deionized water added up to a total volume of 10 μL. PCR cycling conditions were as follows: initial denaturation at 95°C for 4 min, and then 40 cycles of denaturation at 94°C for 30 s, annealing at 60°C for 1 min, and extension at 72°C for 30 s, followed by a final extension at 72°C for 10 min. The amplifications were carried out using the 7900HT Fast Real-Time PCR System (Applied Biosystems). Data acquisitions and analysis were performed using SDS v2.3 Allelic Discrimination Software (Applied Biosystems, Foster City, CA, USA). Direct sequencing of PCR products for two cases of each genotype, which was detected by BigDye Terminator v3.1 Cycle Sequencing Kit and ABI 3130 genetic analyzer (Applied Biosystems), was performed to confirm the genotyping accuracy.

**Table 1:** Primer sequences for genotyping the single nucleotide polymorphisms of *RAC1* gene.

SNPs	Mutation	Area	Forward primer	Reverse primer
rs836488	T>C	Intron 1	5'-AATAGTGTTGTTTTGGTGTTGGTTG-3'	5'-TCCTGATGCTCCTCCCACTTAG-3'
rs702482	A>T	Intron 1	5'-AAAAGTTTGGAGTTGGGCTAAGT-3'	5'-AGACATGATAAAGCAAATACAGCAA-3'
rs10951982	G>A	Intron 1	5'-ATGGCAAAACCCTGTCTCTACTG-3'	'-GAAACGAACATGAGTCGGCTG-3'
rs702483	A>G	Intron 2	5'-TCCTGGAGAATATATCCCTACTGTG-3'	5'-GCCTCAGTCTCCCAAAGTGC-3'
rs6954996	G>A	Intron 5	5'-CAGTGGAGATAATAGCGGCAGAC-3'	5'-TCCTTCACCTAAATCACACCCAG-3'
rs9374	G>A	UTR3’	5'-CTCGTTCTTGGTCCTGTCCCT-3'	5'-GCTGCTACGCTCACTCCATTAC-3'

### Statistical analysis

Differences in the distributions of demographic characteristics between cases and controls were evaluated using the chi-square test for categorical variables and Student’s t-test for continuous variables. Allele and genotype frequencies for the six SNPs in *RAC1* gene were calculated by direct counting. All the SNPs were tested for significant deviation from Hardy-Weinberg equilibrium (HWE) among the controls using goodness-of-fit chi-square test. To avoid the assumption of genetic models, co-dominant (heterozygotes versus major allele homozygotes or minor allele homozygotes versus major allele homozygotes), dominant model (heterozygotes plus minor allele homozygotes versus major allele homozygotes), recessive model (minor allele homozygotes versus major allele homozygotes plus heterozygotes) and additive model (minor allele homozygotes versus heterozygotes versus major allele homozygotes) were all analyzed. Linkage disequilibrium analysis between the different pairs of SNPs was performed using Haploview version 4.2 software package (Broad Institute of Massachusetts Institute of Technology and Harvard University, Cambridge, MA). Haplotype frequency estimation and association analysis were performed with SNPStats software (http://bioinfo.iconcologia.net/SNPStats_web) [33]. The associations between the case-control status and *RAC1* genotypes/halotypes were estimated by computing the crude and adjusted odds rations (ORs) and their corresponding 95% confidence intervals (95% CI) using logistic regression analysis without and with adjustment for possible confounders, including age, gender, smoking status, and drinking status. Additional stratified analysis of the associations of *RAC1* genetic polymorphisms with ESRD risk by subgroups of diabetes status, hypertension status, and smoking status were also performed. All statistical analyses were carried out using SPSS version 17.0 (Chicago, Illinois, USA), and statistical significance level was set at *P* < 0.05 for all analyses.

## Results

### Baseline characteristics of the subjects

The demographic characteristics of the studied subjects are summarized in Table 2. There were no significant differences between cases and controls in gender, age, history of alcohol consumption, and smoking status which indicated that the populations were adequately matched (*P* > 0.05).

**Table 2:** The demographic characteristics of cases and controls.

Characteristics	Renal transplantation (cases)	Healthy subjects (controls)	*P*
**No**.	300	998	
**Gender (*%*)**			0.18
Male	212 (70.7%)	663 (66.4%)	
Female	88 (29.3%)	335 (33.6%)	
**Age (year)**			0.75
mean ± SD	40.44 ± 10.96	39.73 ± 10.81	
**Smoking (*%*)**			0.94
Yes	96 (32.0%)	323 (32.4%)	
No	204 (68.0%)	675 (67.6%)	
**Drinking(*%*)**			0.77
Yes	81 (27.0%)	280 (28.1%)	
No	219 (73.0%)	718 (71.9%)	
**Diabetes (*%*)**			**<0.001**
Yes	64 (21.3%)	0 (0%)	
No	236 (78.7%)	998 (100%)	
**Hypertension (*%*)**			**<0.001**
Yes	143 (47.7%)	0 (0%)	
No	157 (52.3%)	998 (100%)	

### Genotyping and identification of *RAC1* gene polymorphisms

**Fig 2:**
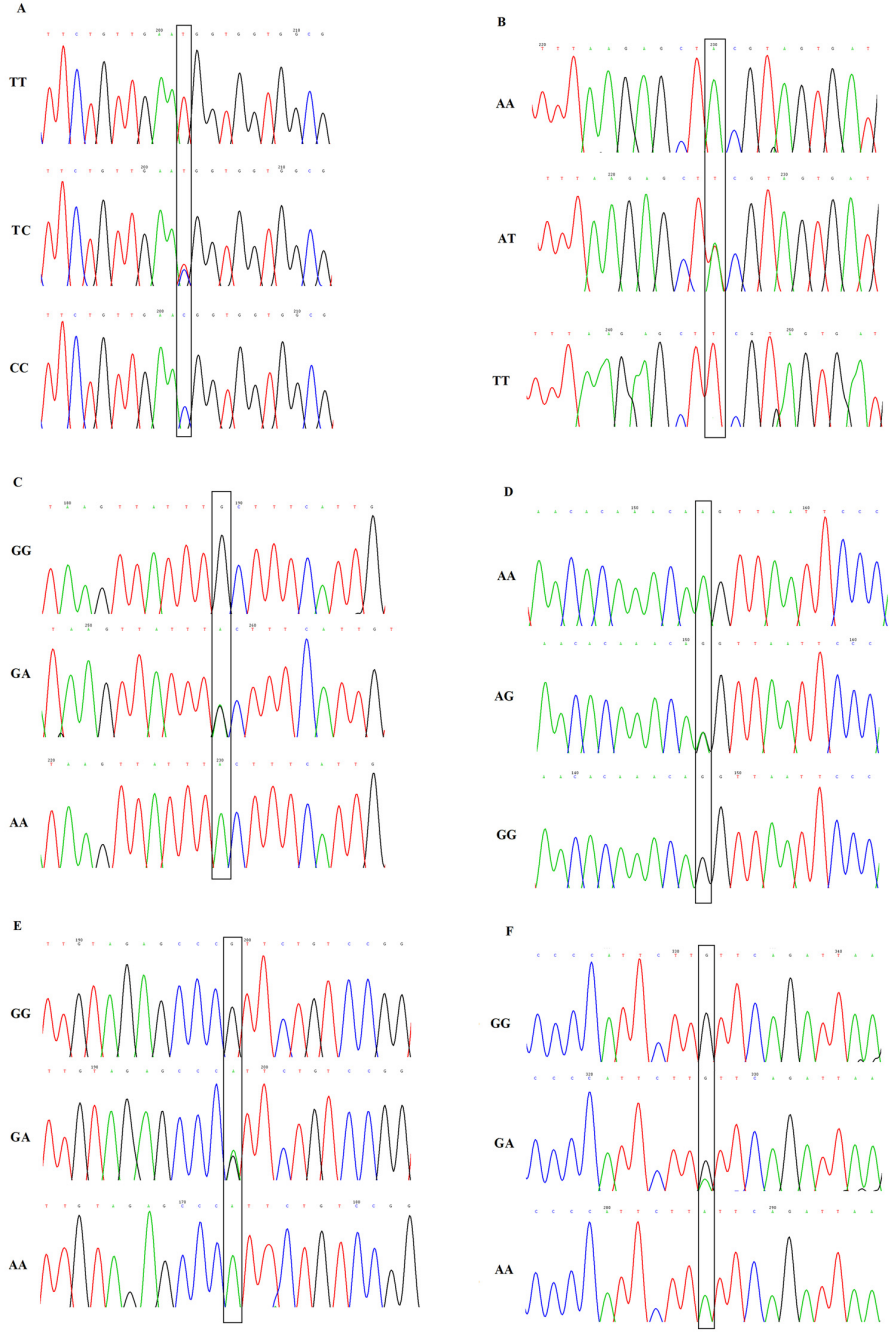
Representative figures of direct sequencing for the six SNPs of *RAC1* gene. (A) rs836488, (B) rs702482, (C) rs10951982, (D) rs702483, (E) rs6954996, and (F) rs9374. Allelic variants were indicated by boxes.

The six selected SNPs of *RAC1* gene in the renal transplant recipients and healthy Chinese subjects were successfully genotyped using TaqMan technology, and the genotyping were found to be consistent with those of direct sequencing. Three different sequencing figures of each SNP are displayed in Fig 2, which represented the 3 genotypes for major allele homozygote, heterozygote, and minor allele homozygote, respectively.

### Distribution of the genotype and allele frequencies

The genotype and allele frequencies of the six SNPs (rs836488, rs702482, rs10951982, rs702483, rs6954996, and rs9374) in cases and controls are presented in Table 3. The genotype distributions for all the tested SNPs in *RAC1* gene among controls were consistent with Hardy-Weinberg equilibrium (*P* > 0.05). As shown in Table 3, the individual SNP analyses revealed that the genotype and allele frequencies of the three SNPs (rs836488, rs702482, and rs702483) were significantly different between the cases and controls. We found that the minor alleles of rs836488, rs702482, and rs702483 showed significant associations with increased risks of ESRD (*P* = 0.019, OR = 1.25, 95% CI = 1.04–1.50 for rs836488; *P* = 0.037, OR = 1.21, 95% CI = 1.01–1.46 rs702482; *P* = 0.030, OR = 1.35, 95% CI = 1.03–1.77 rs702483, respectively). Compared with the major allele homozygotes, the minor allele homozygotes of rs836488 CC, rs702482 TT, and rs702483 GG were also significantly associated with increased ESRD risk (*P* = 0.021, OR = 1.53, 95% CI = 1.07–2.21 for rs836488; *P* = 0.038, OR = 1.47, 95% CI = 1.02–2.11 for rs702482; *P* = 0.006, OR = 3.95, 95% CI = 1.51–10.36 for rs702483, respectively).

**Table 3:** Genotype and allele distribution of *RAC1* gene in renal transplant recipients and healthy subjects.

SNPs	Cases	Controls	Crude OR (95% CI)	*P* value	Adjusted OR (95% CI) ^a^	*P* value^a^
	(*n* = 300)	(*n* = 998)				
	(%)	*n* (%)				
**rs836488**				0.067		
TT	76 (25.3)	313 (31.4)	1.00 (reference)		1.00 (reference)	
TC	148 (49.3)	481 (48.2)	1.27 (0.93–1.73)	0.14	1.27 (0.93–1.74)	0.13
CC	76 (25.3)	204 (20.4)	**1.53 (1.07–2.21)**	**0.021**	**1.53 (1.07–2.21)**	**0.021**
C allele	300 (50.0)	889 (44.5)	**1.25 (1.04–1.50)**	**0.019**	**1.25 (1.04–1.50)**	**0.018**
HWE *P*		0.44				
**rs702482**				0.12		
AA	77 (25.7)	306 (30.7)	1.00 (reference)		1.00 (reference)	
AT	148 (49.3)	489 (49.0)	1.20 (0.88–1.64)	0.24	1.21 (0.89–1.66)	0.23
TT	75 (25.0)	203 (20.3)	**1.47(1.02–2.11)**	**0.038**	**1.47 (1.02–2.11)**	**0.040**
T allele	298 (49.7)	895 (44.8)	**1.21 (1.01–1.46)**	**0.037**	**1.22 (1.01–1.46)**	**0.035**
HWE *P*		0.76				
**rs10951982**				0.31		
GG	172 (57.3)	616 (61.7)	1.00 (reference)		1.00 (reference)	
GA	110 (36.7)	337 (33.8)	1.17(0.89–1.54)	0.26	1.16 (0.89–1.53)	0.29
AA	18 (6.0)	45 (4.5)	1.43 (0.81–2.54)	0.22	1.42 (0.80–2.52)	0.23
A allele	146 (24.3)	427 (21.4)	1.18 (0.95–1.47)	0.13	1.18 (0.95–1.46)	0.14
HWE P		0.90				
**rs702483**				**0.013**		
AA	226 (75.3)	794 (79.6)	1.00 (reference)		1.00 (reference)	
AG	65 (21.7)	196 (19.6)	1.17 (0.85–1.60)	0.35	1.17 (0.85–1.61)	0.33
GG	9 (3.0)	8 (0.8)	**3.95 (1.51–10.36)**	**0.006**	**3.86 (1.47–10.16)**	**0.006**
G allele	83 (13.8)	212 (10.6)	**1.35 (1.03–1.77)**	**0.030**	**1.36 (1.03–1.78)**	**0.029**
HWE *P*		0.28				
**rs6954996**				0.87		
GG	246 (82.0)	824 (82.6)	1.00 (reference)		1.00 (reference)	
GA	51 (17.0)	166 (16.6)	1.03 (0.73–1.45)	0.87	1.04 (0.74–1.47)	0.82
AA	3 (1.0)	8 (0.8)	1.26 (0.33–4.77)	0.72	1.21 (0.32–4.62)	0.78
A allele	57 (9.5)	182 (9.1)	1.05 (0.77–1.43)	0.78	1.05 (0.77–1.44)	0.75
HWE P		0.91				
**rs9374**				0.38		
GG	173 (57.7)	620 (62.1)	1.00 (reference)		1.00 (reference)	
GA	110 (36.7)	327 (32.8)	1.21 (0.92–1.59)	0.18	1.20 (0.91–1.58)	0.19
AA	17 (5.7)	51 (5.1)	1.20 (0.67–2.12)	0.54	1.19 (0.67–2.11)	0.56
A allele	144 (24.0)	429 (21.5)	1.15 (0.93–1.43)	0.19	1.15 (0.93–1.43)	0.21
HWE *P*		0.36				

^a^ Adjusted for age, gender, smoking and drinking.

The results of association analysis under the three different genetic models (additive, dominant, and recessive) were summarized in Table 4. Significant association with increased risk of ESRD was consistently observed for rs836488, rs702482, and rs702483 under additive model (*P* = 0.020, OR = 1.24, 95% CI = 1.03–1.49 for rs836488; *P* = 0.039, OR = 1.21, 95% CI = 1.01–1.45 for rs702482; *P* = 0.031, OR = 1.35, 95% CI = 1.03–1.77 for rs702483, respectively). In addition, similar association was also found in the dominant model for rs836488 (*P* = 0.046, OR = 1.35, 95% CI = 1.01–1.80) and in the recessive model for rs702483 (*P* = 0.007, OR = 3.82, 95% CI = 1.46–10.01). These associations remained significant (*P* < 0.05) after adjusting for traditional risk factors, including age, sex, smoking and drinking statuses. As for the other three SNPs (rs10951982, rs6954996, and rs9374), no significant differences were observed in all comparison models between cases and healthy controls before and after adjusting for confounders (*P* > 0.05).

**Table 4:** Analysis of the six SNPs based on three genetic models.

SNPs	Crude OR (95% CI)	*P* value	Adjusted OR (95% CI) ^a^	*P* value^a^
**rs836488**				
Additive model	**1.24 (1.03–1.49)**	**0.020**	**1.24 (1.04–1.49)**	**0.019**
Dominant model	**1.35 (1.01–1.80)**	**0.046**	**1.35 (1.01–1.81)**	**0.045**
Recessive model	1.32(0.98–1.79)	0.071	1.33 (0.98–1.80)	0.068
**rs702482**				
Additive model	**1.21 (1.01–1.45)**	**0.039**	**1.22 (1.01–1.46)**	**0.036**
Dominant model	1.28 (0.96–1.72)	0.096	1.29 (0.91–1.73)	0.090
Recessive model	1.31 (0.96–1.77)	0.085	1.31 (0.97–1.78)	0.081
**rs10951982**				
Additive model	1.19 (0.95–1.47)	0.13	1.18 (0.95–1.46)	0.14
Dominant model	1.20 (0.93–1.56)	0.17	1.19 (0.92–1.55)	0.19
Recessive model	1.35 (0.77–2.37)	0.29	1.35 (0.77–2.37)	0.30
**rs702483**				
Additive model	**1.35 (1.03–1.77)**	**0.031**	**1.35 (1.03–1.78)**	**0.029**
Dominant model	1.27 (0.90–1.73)	0.12	1.28 (0.94–1.74)	0.11
Recessive model	**3.82 (1.46–10.01)**	**0.007**	**3.76 (1.43–9.85)**	**0.007**
**rs6954996**				
Additive model	1.05 (0.77–1.43)	0.78	1.05 (0.77–1.44)	0.75
Dominant model	1.04 (0.74–1.46)	0.82	1.05 (0.75–1.47)	0.78
Recessive model	1.25 (0.33–4.74)	0.72	1.22 (0.32–4.63)	0.77
**rs9374**				
Additive model	1.15 (0.93–1.42)	0.20	1.15 (0.93–1.42)	0.21
Dominant model	1.20 (0.93–1.57)	0.17	1.20 (0.92–1.56)	0.18
Recessive model	1.12 (0.63–1.96)	0.70	1.11 (0.63–1.96)	0.71

^a^ Adjusted for age, gender, smoking and drinking.

Furthermore, we investigated the effect of *RAC1* SNPs on ERSD risk according to smoking status, diabetes status, and hypertension status (Table 5). In non-smokers, similar significant associations were found between the cases and controls for rs836488, rs702482, and rs702483 under minor homozygotes versus major homozygotes co-dominant model (*P* = 0.029, OR = 1.64, 95% CI = 1.05–2.55 for rs836488; *P* = 0.047, OR = 1.57, 95% CI = 1.01–2.45 for rs702482; *P* = 0.001, OR = 7.23, 95% CI = 2.14–24.45 for rs702483, respectively), additive model (*P* = 0.028, OR = 1.28, 95% CI = 1.03–1.59 for rs836488; *P* = 0.045, OR = 1.25, 95% CI = 1.01–1.56 for rs702482; *P* = 0.004, OR = 1.61, 95% CI = 1.17–2.24 for rs702483, respectively), dominant model (*P* = 0.036, OR = 1.46, 95% CI = 1.03–2.08 for rs836488; *P* = 0.029, OR = 1.50, 95% CI = 1.04–2.17 for rs702483), and recessive model (*P* = 0.002, OR = 6.80, 95% CI = 2.02–22.94 for rs702483). In smokers, however, no statistically significant association was observed. After stratification analysis under the three genetic models, the results from non-diabetics and non-hypertensive subgroups were consistent with the overall analysis as expected.

**Table 5:** Stratification analysis of *RAC1* polymorphisms in renal transplant recipients and healthy subjects.

Variables	SNPs	Cases ^a^	Controls ^a^	Additive model		Dominant model		Recessive model	
				OR (95% CI) ^a^	*P* ^a^	OR (95% CI) ^b^	*P* ^b^	OR (95% CI) ^b^	*P* ^b^
Non-diabetics	rs836488	59/117/60	313/481/204	**1.25 (1.02–1.53)**	**0.029**	**1.39 (1.01–1.93)**	**0.047**	1.31 (0.94–1.83)	0.11
	rs702482	59/118/59	306/489/203	**1.23 (1.01–1.51)**	**0.044**	1.35 (0.97–1.87)	0.074	1.29 (0.92–1.81)	0.13
	rs10951982	136/84/16	616/337/45	1.18 (0.93–1.49)	0.18	1.17 (0.87–1.56)	0.29	1.48 (0.82–2.69)	0.20
	rs702483	177/52/7	794/196/8	**1.38 (1.02–1.86)**	**0.038**	1.31 (0.93–1.83)	0.12	**3.68 (1.31–10.35)**	**0.014**
	rs6954996	193/40/3	824/166/8	1.10 (0.78–1.55)	0.57	1.09 (0.75–1.58)	0.65	1.50 (0.39–5.78)	0.56
	rs9374	135/86/15	620/327/51	1.16 (0.92–1.47)	0.210	1.20 (0.90–1.61)	0.21	1.21 (0.66–2.20)	0.54
Non-hypertensive	rs836488	37/77/43	313/481/204	**1.33 (1.05–1.69)**	**0.020**	**1.50 (1.01–2.24)**	**0.046**	1.43 (0.97–2.11)	0.072
	rs702482	36/79/42	306/489/203	**1.32 (1.04–1.68)**	**0.025**	**1.50 (1.00–2.25)**	**0.049**	1.41 (0.95–2.08)	0.089
	rs10951982	88/57/12	616/337/45	1.25 (0.95–1.65)	0.12	1.24 (0.88–1.75)	0.23	1.68 (0.86–3.31)	0.13
	rs702483	115/37/5	794/196/8	**1.50 (1.05–2.13)**	**0.025**	1.42 (0.96–2.10)	0.079	**4.28 (1.34–13.66)**	**0.014**
	rs6954996	132/23/2	824/166/8	0.96 (0.63–1.48)	0.86	0.92 (0.58–1.47)	0.73	1.61 (0.33–7.88)	0.56
	rs9374	86/59/12	620/327/51	1.27 (0.97–1.67)	0.084	1.32 (0.94–1.86)	0.12	1.48 (0.76–2.88)	0.26
Non-smoking	rs836488	52/101/51	227/310/137	**1.28 (1.03–1.59)**	**0.028**	**1.46 (1.03–2.08)**	**0.036**	1.32 (0.91–1.91)	0.14
	rs702482	52/102/50	222/315/138	**1.25 (1.01–1.56)**	**0.045**	1.42 (0.99–2.02)	0.054	1.29 (0.89–1.87)	0.18
	rs10951982	122/70/12	425/216/34	1.12 (0.86–1.45)	0.40	1.14 (0.82–1.57)	0.43	1.20 (0.61–2.36)	0.61
	rs702483	150/46/8	546/125/4	**1.61 (1.17–2.24)**	**0.004**	**1.50 (1.04–2.17)**	**0.029**	**6.80 (2.02–22.94)**	**0.002**
	rs6954996	165/38/1	563/107/5	1.15 (0.79–1.69)	0.46	1.19 (0.80–1.79)	0.39	0.67 (0.08–5.75)	0.71
	rs9374	123/70/11	413/212/40	1.06(0.81–1.36)	0.71	1.10 (0.80–1.52)	0.57	0.92 (0.46–1.84)	0.82
Smoking	rs836488	24/47/25	86/171/66	1.19 (0.86–1.66)	0.30	1.16 (0.69–1.98)	0.58	1.37 (0.80–2.34)	0.25
	rs702482	25/46/25	84/174/65	1.16 (0.83–1.61)	0.39	1.07 (0.63–1.82)	0.79	1.38 (0.81–2.35)	0.24
	rs10951982	50/40/6	191/121/11	1.30 (0.88–1.93)	0.18	1.32 (0.83–2.10)	0.24	1.70 (0.60–4.82)	0.32
	rs702483	76/19/1	248/71/4	0.88 (0.53–1.49)	0.64	0.87 (0.50–1.52)	0.62	0.72 (0.08–6.64)	0.77
	rs6954996	81/13/2	261/59/3	0.94 (0.53–1.64)	0.82	0.83 (0.45–1.55)	0.56	2.86 (0.46–17.94)	0.26
	rs9374	50/40/6	123/70/11	1.37 (0.93–2.02)	0.11	1.42(0.89–2.25)	0.14	1.71 (0.60–4.83)	0.31

^a^ Major homozygotes/heterozygotes/minor homozygotes

^b^ Adjusted for age, gender, smoking and drinking (besides stratified factors accordingly).

### Linkage disequilibrium and haplotype analysis

**Fig 3:**
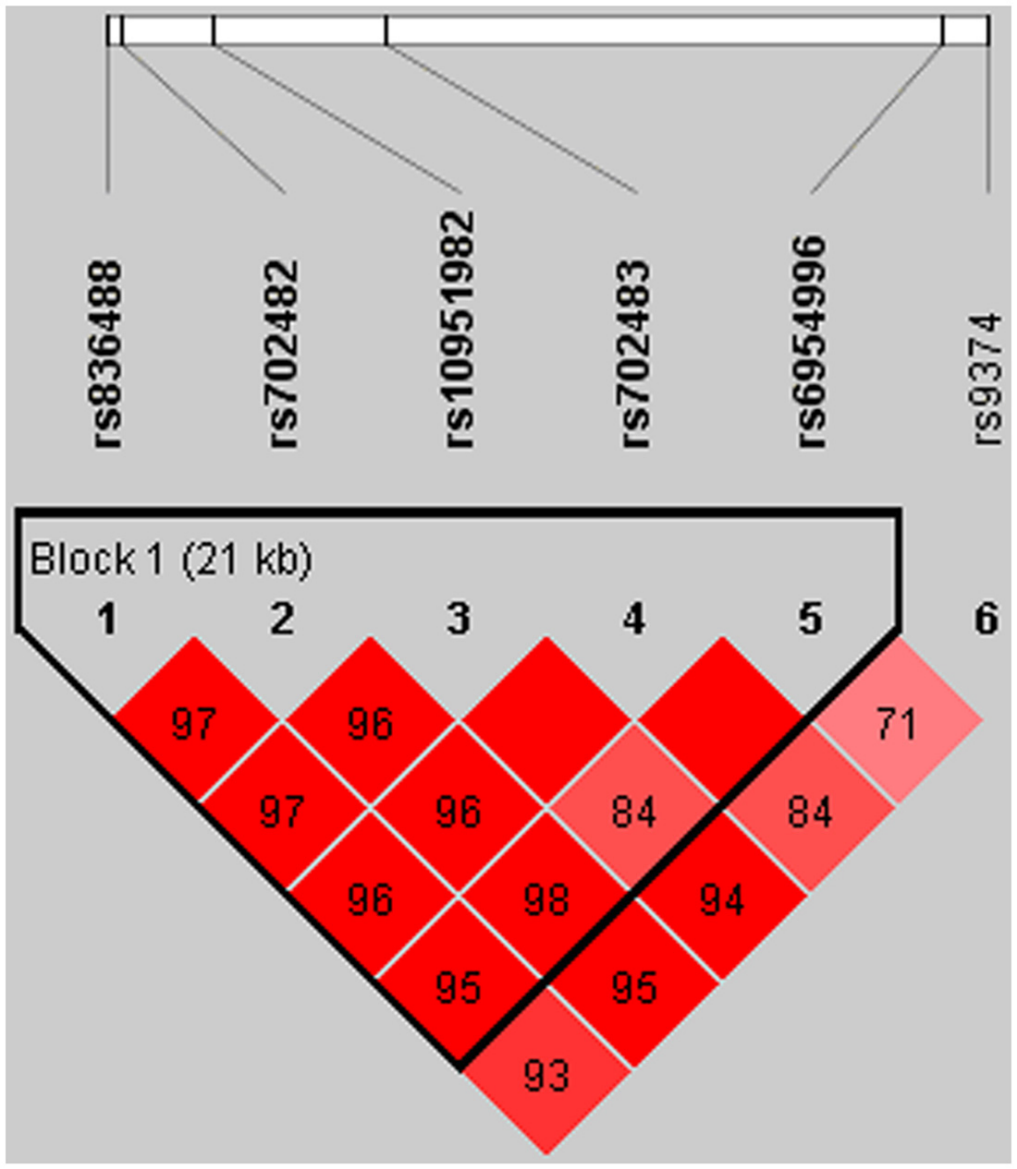
Linkage disequilibrium results among the six SNPs in *RAC1* gene.

We then performed the linkage disequilibrium (LD) and haplotype analysis among these six SNPs. The LD structure between each pair of SNPs in *RAC1* gene is shown in Fig 3. The results indicated that the six *RAC1* SNPs (rs836488, rs702482, rs10951982, rs702483, rs6954996, and rs9374) were in high LD, and the lowest D’ was greater than 0.80. The frequencies of derived haplotypes (≥ 1%) from these six *RAC1* SNPs are presented in Table 6. Twenty-eight haplotypes of the six SNPs were inferred, five of which had frequency greater than 0.01. The most common haplotype was "T-A-G-A-G-G", which accounted for 48.92% of cases and 53.89% of controls. As compared to the most common haplotype, only "C-T-G-G-G-G" haplotype was significantly associated with increased risk of ESRD (*P* = 0.011, OR = 1.46, 95% CI = 1.09–1.94). In addition, it would be noted that "C-T-A-A-G-A" (*P* = 0.073, OR = 1.24, 95% CI = 0.98–1.56) showed a borderline significant association with increased risk of ESRD, on the other hand, haplotype "C-T-G-A-A-G" showed a marginal significant association with decreased risk of ESRD (*P* = 0.072, OR = 0.57, 95% CI = 0.31–1.05). As shown in Table 5, a significant difference in the distribution of the global haplotypes between the cases and controls (*P* = 0.015) was also found.

**Table 6:** Frequencies of derived haplotypes (≥1%) from six examined polymorphisms of *RAC1* gene in cases and controls.

Haplotype ^a^	Cases (%)	Controls (%)	Crude OR (95% CI)	*P* value	Adjusted OR (95% CI) ^b^	*P* value ^b^
T-A-G-A-G-G	48.92	53.89	1.00 (reference)		1.00 (reference)	
C-T-A-A-G-A	22.99	20.15	1.24 (0.98–1.56)	0.073	1.24 (0.98–1.56)	0.075
C-T-G-G-G-G	13.53	10.00	**1.46 (1.09–1.94)**	**0.011**	**1.46 (1.09–1.95)**	**0.011**
C-T-G-A-A-G	9.50	8.58	1.21 (0.87–1.68)	0.25	1.22 (0.88–1.69)	0.24
C-T-G-A-G-G	2.19	4.18	0.57 (0.31–1.05)	0.072	0.58 (0.32–1.07)	0.080
Global				0.015		0.016

^**a**^ Alleles incorporated on a haplotype were in order of rs836488, rs702482, rs10951982, rs702483, rs6954996, and rs9374 in *RAC1* gene.

^**b**^ Adjusted for age, gender, smoking and drinking.

## Discussion

*RAC1* gene could influence susceptibility to renal failure by altering the activity and expression of Rac1 which is the member of the Rho family of small GTP-binding proteins [27, 30]. We selected 6 SNPs in *RAC1* gene and conducted an association study in Chinese Han population. To our knowledge, this study for the first time demonstrated that SNP rs702483 in *RAC1* gene was distributed differentially between the renal transplant recipients and healthy subjects. Furthermore, it was also shown that the allele distributions of rs836488, rs702482, and rs702483 in *RAC1* gene were different between the two groups. Based on our results, therefore, it is reasonable to speculate that polymorphisms in the *RAC1* gene might be associated with risk factors for ESRD.

In single-locus analyses, individuals with minor homozygotes CC, TT, and GG would have more risk to develop ESRD as compared to those with major homozygote TT in rs836488, AA in rs702482, and AA in rs702483, respectively. When compared the minor homozygote with major homozygote and heterozygote, individuals with minor homozygote GG in rs702483 showed a tendency for increased risk of ESRD. Compared with minor homozygote and heterozygote, individuals with major homozygote CC in rs836488, were found to carry lower risk of ESRD. In the additive model, it was found that allele C in rs836488, allele T in rs702482, or allele G in rs702483 might increase the risk of ESRD as compare to allele T in rs836488, allele A in rs702482, or allele A in rs702483, respectively.

The pairwise LD and haplotype distribution analyses showed that six *RAC1* SNPs were in LD and in one haplotype block. According to the haplotypes association analysis, it was found that individuals carrying the "C-T-G-G-G-G" haplotype might have higher risk of developing ESRD as compared to the most common haplotype "T-A-G-A-G-G". Considering the co-effect of *RAC1* gene, several confounding factors were adjusted in the association analysis. No substantial impact on the risk estimates was found after adjusting for age, gender, smoking, and drinking. In addition, compared the results in our study with reported data in the HapMap databases (www.hapmap.ncbi.nlm.nih.gov), the genotypic and allelic frequencies of all the six examined SNPs were found to be similar to those of the Chinese and other Asian population as expected.

Cigarette smoking is considered to be an important risk factor for renal damage; therefore, we further explored the possible interactions of *RAC1* gene polymorphisms with smoking. Our recruited cohort was divided into smoking and non-smoking groups, and it was observed that the effects of *RAC1* gene polymorphisms on the risk of ESRD differed according to the smoking status. In the non-smoking group, a significant association was observed between the three SNPs (rs836488, rs702482, and rs702483) of *RAC1* gene and increased ESRD risks, which was similar to the overall cohort we recruited. However, such significant association was not consistently found for rs836488, rs702482, and rs702483 in the smoking group. These results indicated a possible existence of gene-smoking interaction in modulating the risk of ESRD.

The mechanisms underlying the differential association of *RAC1* SNPs variants with the risk for ESRD remain unclear. Some studies have indicated that *RAC1* gene has a critical role in the regulation of the gene expression involved in Rac1 activation and mineralocorticoid-receptor activation, which would be associated with the renal failure [29, 30]. Aleixo *et al*. showed that carriage of the risk allele resulted in increased Rac1 activity and gene expression [25], leading to increased proinflammatory cytokine expression and subsequent activation of Rac1, which are involved in podocyte foot process effacement and proteinuric renal failure according to the motile podocyte hypothesis. In a cross-sectional study, Alejandra *et al*. found that SNPs rs10951982 and rs836478 in the *RAC1* gene were correlated with some clinical and biochemical parameters, including blood pressure, metalloproteinase-9 (MMP-9), interleukin 6 (IL-6), and plasminogen activator inhibitor-1 (PAI-1) [27], which played an important role in the development and progression of organ damage, e.g. renal injury. Those findings indirectly support our speculation that *RAC1* gene polymorphisms might increase the possibility of being associated with the susceptibility of ESRD.

Furthermore, Bourgine *et al*. addressed that polymorphism of *RAC1* gene can be regarded as a possible additional mechanism that might contribute to the inter-individual differences in the therapeutic effect of thiopurine drugs [32], which are widely applied for the treatment of inflammatory bowel diseases (IBD) [34]. They found that functional genetic polymorphisms of *RAC1*, which were located in the regions involved in an mRNA splicing process and modified the amino acid sequence of Rac1, affected its expression and modulated the risk of adverse drug reaction in patients with IBD under thiopurine treatment. It is well known that thiopurine drugs can be used as an immunosuppressive agent in organ transplantations, e.g. renal transplantation. Moreover, previous researches have illustrated that thiopurine drugs exerts its immunosuppressive activity by blockade of Rac1 activation, suppression of Rac1 functions on T-cell survival and T-cell-APC conjugation, and regulation of Rac1 activity. Therefore, it is reasonable to suggest that attention should be paid to the adverse drug reaction in organ transplantations with thiopurine treatment.

In this study, it is difficult to conclude the exact contribution of *RAC1* gene polymorphisms on ESRD particularly because of its complicated mechanisms and possibly because Rac1 is expressed in various cell types and has pivotal and divergent functions in numerous cellular pathways. In addition, given many comorbidities in renal transplant recipients which could be also involved by Rac1 due to its broad range of associations, these observed findings might be affected by survival bias. Therefore, it is necessary to make further studies on the divergent functions of Rac1 to confirm whether these three SNPs are causally related to ESRD and for deeply understanding the association of *RAC1* gene polymorphisms with ESRD.

## Limitations

Some limitations in this study need to be addressed. Firstly, the possibility of selection bias of subjects could not be excluded in this hospital-based case-control study. However, the genotype frequencies of all the SNPs in patients and normal controls were aligned with HWE, suggesting that the selection bias was unlikely to substantially influence the results. Secondly, although we had a relative large sample size of 300 patients with renal transplants and 998 controls, this sample size might still be insufficient to determine the genetic associations, particularly for rare alleles. Additionally, an important consideration for the genetic association studies is false positive results arising from multiple testing. Owing to the high degree of correlation between the six tested SNPs, the overly conservative Bonferroni correction was not appropriate in this study, but our findings should be interpreted with caution. Thirdly, only gene polymorphism analysis was performed in this present study without addressing the mRNA and protein expressions differences in renal tissues from these transplant recipients and controls. Therefore, we cannot postulate that these polymorphisms could have functional role. Further studies are required to explore the functional relevance of these SNPs and clarify the underlying molecular mechanisms for the observed associations. Fourthly, we did not obtain detailed information on the underlying primary renal disease in patients with renal transplants, which restricted further association analyses of the genetic effects of Rac1 with respect to renal diseases factors, such as inflammation and autoimmune disorders. Finally, our included subjects were restricted to individuals of Chinese Han population and the results from our study were not replicated, and therefore our observations cannot be extrapolated to other races and ethnicities. Nevertheless, our findings might provide interesting information and new insights into the genetics of ESRD.

## Conclusions

In summary, this study for the first time reported the frequency and distribution of the six SNPs (rs836488, rs702482, rs10951982, rs702483, rs6954996, and rs9374) of *RAC1* gene in patients with renal transplantations and healthy subjects in China. It was indicate that rs836488, rs702482, and rs702483 in *RAC1* gene were distributed differentially between the renal transplant recipients and healthy subjects. These *RAC1* SNPs might serve as susceptible genetic marker for ESRD in the Chinese Han Population. Further studies with larger sample size and in diverse ethnic populations are required to replicate our findings, and functional studies are necessary to elucidate the potentially biological mechanisms.
